# Participant recruitment and retention in longitudinal preconception randomized trials: lessons learnt from the Calcium And Pre-eclampsia (CAP) trial

**DOI:** 10.1186/s13063-017-2220-0

**Published:** 2017-10-26

**Authors:** Theresa A. Lawrie, Ana Pilar Betrán, Mandisa Singata-Madliki, Alvaro Ciganda, G. Justus Hofmeyr, José M. Belizán, Tina Dannemann Purnat, Sarah Manyame, Catherine Parker, Gabriela Cormick, on behalf of the Calcium and Pre-eclampsia Study Group

**Affiliations:** 1 Effective Care Research Unit, Eastern Cape Department of Health/Universities of the Witwatersrand, Walter Sisulu and Fort Hare, East London, South Africa; 2 HRP – UNDP/UNFPA/UNICEF/WHO/World Bank Special Programme of Research, Development and Research Training in Human Reproduction, Department of Reproductive Health and Research, World Health Organization, Geneva, Switzerland; 3 Department of Mother and Child Health Research, Institute for Clinical Effectiveness and Health Policy (IECS), Emilio Ravignani 2024, Buenos Aires, Argentina; 4 Division of Information, Evidence, Research and Innovation, World Health Organization Regional Office for Europe, Copenhagen, Denmark; 5 University of Zimbabwe College of Health Sciences, Harare, Zimbabwe

**Keywords:** Calcium, Pre-eclampsia, Preconception, Recruitment, Retention, Randomized

## Abstract

**Background:**

The preconception period has the potential to influence pregnancy outcomes and randomized controlled trials (RCTs) are needed to evaluate a variety of potentially beneficial preconception interventions. However, RCTs commencing before pregnancy have significant participant recruitment and retention challenges. The Calcium And Pre-eclampsia trial (CAP trial) is a World Health Organization multi-country RCT of calcium supplementation commenced before pregnancy to prevent recurrent pre-eclampsia in which non-pregnant participants are recruited and followed up until childbirth. This sub-study explores recruitment methods and preconception retention of participants of the CAP trial to inform future trials.

**Methods:**

Recruiters at the study sites in Argentina, South Africa and Zimbabwe completed post-recruitment phase questionnaires on recruitment methods used. Qualitative data from these questionnaires and quantitative data on pre-pregnancy trial visit attendance and pregnancy rates up to September 2016 are reported in this paper. RStudio (Version 0.99.903 https://www.rstudio.org) statistical software was used for summary statistics.

**Results:**

Between July 2011 and 8 September 2016, 1354 women with previous pre-eclampsia were recruited. Recruitment took 2 years longer than expected and was facilitated mainly through medical record/register and maternity ward/clinic-based strategies. Recruiters highlighted difficulties associated with inadequate medical records, redundant patient contact details, and follow-up of temporarily ineligible women as some of the challenges faced. Whilst the attendance rates at pre-pregnancy visits were high (78% or more), visits often occurred later than scheduled. Forty-five percent of participants became pregnant (614/1354), 33.5% (454/1354) within 1 year of randomization.

**Conclusions:**

In preconception trials, both retrospective and prospective methods are useful for recruiting eligible women with certain conditions. However, these are time-consuming in low-resource settings with suboptimal medical records and other challenges. Trial planners should ensure that trial budgets cover sufficient on-site researchers with pre-trial training, and should consider using mobile phone and web-based electronic tools to optimize recruitment and retention. This should lead to greater efficiency and shorter trial durations.

**Trial registration:**

Pan-African Clinical Trials Registry, Registration Number: PACTR201105000267371. The trial was registered on 6 December 2016.

## Background

Preconception is now recognized as a period with the potential to influence pregnancy outcomes and long-term child health [1]. Whilst certain interventions recommended during pregnancy might also have an impact if commenced before pregnancy, evidence on preconception interventions is scarce, particularly from low- and middle-income countries (LMICs). Preconception interventions that might influence pregnancy outcomes include interventions targeting chronic conditions such as epilepsy, hypertension, and diabetes [2], nutritional and/or lifestyle interventions for underweight and overweight populations [3], interventions to reduce substance use [4], thromboprophylaxis to reduce pregnancy losses [5, 6], interventions targeting infections such as HIV [7, 8], and interventions to reduce pre-eclampsia [3, 9]. There is, therefore, a need for well-conducted, preconception randomized controlled trials (RCTs) to improve preconception guidance, and various preconception trials are underway [10–12].

However, RCTs are known to have difficulties associated with recruitment, compliance, and retention of participants over extended study periods, which can sometimes lead to early trial closures. In addition, participant attrition can cause methodological problems that influence the study results [13]. Preconception RCTs are arguably the most challenging type of RCT to conduct, as eligible non-pregnant women need to be recruited and retained until conception occurs (if and when), and then further, throughout pregnancy, to delivery or beyond. A variable proportion of randomized (non-pregnant) women, therefore, are not included in the final sample of pregnant women, and time to conception cannot be estimated – unlike time to delivery. Recruitment for preconception RCTs is particularly difficult because so many pregnancies are unplanned and highly eligible women may only access the healthcare system once they are already pregnant. Thus, when planning a preconception RCT it is important to consider strategies to optimize participant recruitment and retention.

We designed a RCT known as the Calcium And Pre-eclampsia (CAP) trial to test the hypothesis that calcium supplementation commenced before pregnancy will reduce the incidence of recurrent pre-eclampsia more effectively than supplementation starting at 20 weeks’ gestation [12]. As with preconception folic acid supplementation [14–19], if preconception calcium supplementation is shown to be effective, it could have important implications for food fortification policies, particularly in countries with low dietary calcium intake [9]. This article describes a sub-study of the multi-country CAP trial, exploring the recruitment methods and participant retention in the preconception phase of this trial including difficulties and challenges experienced.

## Methods

### Objective

The aim of this sub-study was to explore recruitment methods and retention of participants in the CAP trial to inform future preconception trials conducted in LMICs.

### Trial design and participants

The World Health Organization CAP trial is a multi-centre, double-blind, parallel-arm, placebo-controlled randomized trial of long-term calcium supplementation in women at high risk of pre-eclampsia. Non-pregnant women were eligible if they had developed pre-eclampsia in their most recent pregnancy, were planning to become pregnant and were willing to provide written informed consent.

### Setting

This multi-country trial includes one site in Zimbabwe (comprising Harare and Mbuya Nehanda Maternity Hospitals), four sites in South Africa (Frere and Cecilia Makiwane Hospitals in East London, Chris Hani Baragwanath Hospital in Johannesburg, Tygerberg Hospital near Stellenbosch, and Mowbray Maternity Hospital in Cape Town), and a site in Argentina (Institute for Clinical Effectiveness and Health Policy) comprising four hospitals (Hospital Italiano, Hospital San Justo, and the Center of Medical Education and Clinical Investigations in Buenos Aires, and the Institute of Maternity and Gynecology, Nuestra Señora de Mercedes in Tucumán province). Most of these referral hospitals serve mainly low-income populations with low dietary calcium intake.

### Interventions

Participants in the study group received 500 mg of elemental calcium daily (in the form of calcium carbonate) from randomization (preconception) until 20 weeks’ gestation, whereas participants in the control group received identical-looking placebos. All women received unblinded calcium supplementation (1.5 g elemental calcium daily) from 20 weeks’ gestation until delivery as per WHO recommendations for prevention of pre-eclampsia [20].

### Outcomes

The primary outcome of this trial is pre-eclampsia; secondary outcomes include pregnancy, miscarriage, maternal and neonatal complications related to pre-eclampsia, and compliance. For a complete list please refer to the published protocol [12].

### Sample size

The sample size calculation was informed by a previous WHO study of calcium supplementation from 20 weeks’ gestation in which the incidence of hypertension (with or without proteinuria) among relatively low-risk pregnant women in South Africa was 14% [21]. Women with pre-eclampsia in a preceding pregnancy have a very high risk of recurrence, approaching 50% in some studies [22]. Therefore, for the power calculation, we assumed the incidence of pre-eclampsia in our trial, which involves only high-risk women, to be 25%. To show a reduction in pre-eclampsia to 15%, we calculated that we needed 540 participants with pregnancies continuing beyond 20 weeks’ gestation using Epi Info™ software (CDC) (alpha = 5%, beta = 80%). We anticipated that 50% of women recruited would become pregnant during the study. Therefore, allowing for a miscarriage rate of 15% and loss to follow-up of 10%, we calculated that we needed a sample size of approximately 1440 non-pregnant women.

### Study methods

The methods for this double-blind RCT have been described [12]. Various recruitment methods were proposed in the protocol, and study sites customized the methods to their individual settings. Non-pregnant women attending screening and subsequent research clinic visits were offered compensation at each visit for travel expenses.

To facilitate screening and recruitment, the screening form grouped eligibility criteria into two sections according to whether a woman was permanently ineligible (e.g., did not have previous pre-eclampsia) or temporarily ineligible (e.g., not in a sexual relationship). Women in the latter group could be invited for another screening visit at a later stage. Following randomization, participants were required to attend the research clinic visits every 12 weeks for follow-up from preconception through to delivery. Between-visit contact was to be maintained by 4-weekly telephone calls.

Case Report Form data were entered and validated in an online data management system (OpenClinica; www.openclinica.com) by researchers at the sites.

### Sub-study methods

For this exploratory, mixed-methods sub-study, researchers responsible for recruitment at each site were asked in July 2016 to complete a questionnaire on the recruitment methods used and the “pros” and “cons” of each method. Qualitative data compiled from these questionnaires were tabulated. RStudio (Version 0.99.903 https://www.rstudio.org) statistical software was used for summary statistics. For retention calculations using data up to September 2016, the denominator excluded women who became pregnant. As the randomization code has not yet been broken, no comparative data were analyzed.

## Results

Recruitment commenced in July 2011 and was completed on 8 September 2016, taking 2 years longer than anticipated and involving more sites than originally planned.

Table 1 shows the findings of the recruiter-completed questionnaire, highlighting the challenges encountered by different recruitment approaches, lessons learnt, and practical considerations for future trials. Most participants were recruited either by searching past medical records and maternity registers to identify and make contact with potentially eligible women, or by visiting hospital postnatal wards and clinics to approach women who had recently experienced pre-eclampsia. The main advantage of the former approach was the potentially immediate access to large databases of eligible women; the main disadvantage was the huge recruiter workload involved in identifying, pre-screening, and making contact, and the low response and recruitment rates following contact. Medical records and registers often lacked accurate contact details and diagnoses and this contributed to the low recruitment rates with this method. In addition, it required high levels of coordination with local hospital staff not involved in the trial to facilitate access to medical records. As this comprised additional work for hospital staff, without additional compensation, it was not easy for recruiters to implement and sustain.

**Table 1:** Approaches to identify and recruit women for the Calcium And Pre-eclampsia (CAP) trial: advantages, difficulties, and lessons learnt

Recruitment approach (sites)	Advantages	Difficulties	Lessons learnt and recommendations for future action
Retrospective identification and recruitment of women			
Searching laboratory or other computerized hospital records to identify potential participants (based on pre-eclampsia diagnosis) from the previous 5 years, then sourcing their contact details via medical records (all countries)	• Identified eligible women • Eligibility was fairly easy to determine • Large databases • Women were more likely to be planning a pregnancy due to the time elapsed since their previous pregnancy	• Access to medical records was often slow and depended on the goodwill and availability of laboratory and records department staff • Recruiters felt uncomfortable asking staff to assist without financial compensation • Due to poor codification at some sites, computerized hospital records under-reported pre-eclampsia • Many eligible women were unreachable due to missing, incomplete or redundant contact details • Response rates were poor: e.g., “For every 40 calls made, about 2 or 3 women agreed to come in for a screening visit” • Recruitment rates were poor: “…many women were reluctant to say ‘no’ to a request to participate, so they said ‘yes’ but 1 out of every 2 or 3 women did not attend their screening appointments” • Searching and pre-screening was a laborious process • Telephone screening was time-consuming and expensive • Recruiters often made evening telephone calls in efforts to make contact • Some women were identified more than once and received duplicate calls • Cold-calling caused alarm (many women thought the recruiters were debt collectors) and women were uncomfortable answering personal questions, such as family planning intentions, over the telephone with a stranger • Potential participants were finite	• A good source of participants but very laborious and time-consuming • Meet with the medical records/archives department early on and establish the terms of the access, the work involved, and compensation for additional work if appropriate, e.g., agree a day or days on which trial-related work can be performed • Include a hospital staff member in the research team to reduce bureaucratic/logistical difficulties in accessing records and registers • Before starting recruitment, dedicate a recruiter or recruiters to compile a complete list of all potentially eligible and contactable women and schedule all telephonic contacts in an e-calendar • Have a recruiter dedicated to retrospective pre-screening of these women • Have a computer dedicated to identification/recruitment activities • Consider ways of sorting telephonic recruitment, e.g., according to pregnancy outcome, (low) parity, to prioritize women most likely to desire another pregnancy • Check that newly identified women are not already listed, and have a system in place to prevent duplicating telephone calls • Establish a threshold number of calls (e.g., 3 or 4) that, if not answered over a period of trying (e.g., 1 month), further calls are stopped, so as not to harass potentially eligible women who do not wish to participate • Provide recruiters with plenty of mobile phone airtime and ensure at least 1 phone per research staff member
Searching maternity “high-care” ward registers from the previous 5 years, then sourcing medical records and telephone numbers	• Identified eligible women, often with severe PE/E • Eligibility was fairly easy to determine	• Registers were often missing crucial information, e.g., diagnosis, contact details • Recruiters often required assistance from busy ward staff to clarify queries, locate registers, etc., and sometimes felt uncomfortable disturbing them • Searching paper records was a laborious process • Telephone screening was time-consuming and expensive • Sometimes involved recruiters making evening telephone calls in efforts to make contact • As above, women were often alarmed by this cold-calling method • Potential participants were finite	• Establish a good relationship with the “high-care” staff to facilitate access to ward registers • Identify a research “champion” among the ward staff and consider ways to incentivize and keep staff informed of trial progress • Give feedback to departments and encourage in-house training on record keeping
Searching other records and registers, e.g., previous pre-eclampsia study databases, pediatric records	• Identified eligible women • Eligibility was fairly easy to determine	• Many of the same difficulties as above, such as redundant contact details and time-consuming work • Response and recruitment rates were poor	• Probably not a good use of trial resources
Advertising with posters	• Identified eligible women • Respondents were usually interested in participating • Easy to implement • Posters also served to remind clinical staff of the trial	• Women exposed to poster advertising in clinics were usually unwell, pregnant, or requiring contraception, so eligible participants were limited • Hospital records were often not available for women referred from community clinics, which made screening and baseline data collection challenging • Clinic staff had limited availability and recruiters found it challenging to inform them about the trial or interest them in promoting the trial • Posters were removed or damaged in some clinics and needed replacing • Respondents had to bear the initial cost of a telephone call, which may have put some women off responding	• A good supplementary activity but yields may not be high • Budget for design and printing of promotional posters (or advertisements) • Consider ways of incentivizing clinic staff to identify and encourage potentially eligible respondents • Visits clinics at regular intervals to update staff and replace posters if necessary • Give a free-call option or make it clear on the poster that recruiters will call respondents back if they reply by text
Advertising in newspapers	• Respondents were usually interested in participating	• Newspaper advertising was expensive (both to design and print) and response rates were low, possibly because many women do not spend money on newspapers • Pregnant women and other ineligible women often responded • Hospital records were often not available, which made screening and baseline data collection challenging	• Avoid advertising in the general press • Identify and consider other appropriate site-specific media, e.g., “free” local newspapers/magazines that appeal to women • Use of flyers may be more efficient and less expensive as it reaches larger numbers of community women, gives more information • Budget for design and printing of advertisements
Presenting on radio talk-shows	• Identified potentially eligible women in the community • Respondents were usually interested in participating • Wide exposure	• Radio stations were busy and it was difficult to get slots on talk-shows, therefore, this promotional activity was only done once	• Radio advertising, as well as talk-shows, may be a good supplementary strategy if resources allow (ads are repeated, unlike talk-shows, which are usually one-off) • Above the cost of the advert, investigators would need to budget for airtime costs, which vary according to the station, time of day, and length. Alternatively, some radio stations offer “live-reads” by the presenter, which might be more cost-effective
Using LHWs to promote participation through community outreach (door-to-door visits, community clinics)	• Identified potentially eligible women in the community • Fair number of referrals initially • Easy to trace participants recruited this way when they missed appointments because of the LHW contact	• Many ineligible women were referred by LHWs • Transport money and incentives, such as promotional T-shirts and mugs, were provided to LHWs employed by city council clinics; however, it was difficult to keep LHWs incentivized and referrals declined over time • The area covered by each LHW was finite	• Train LHWs and provide checklists for them on which to base referrals • Provide supervision for LHW with regular feedback • Improve incentives for LHWs • Use LHWs to distribute flyers in their communities to increase exposure • Budget for LHW training day, supervision, transport money, and incentives
Prospective identification and recruitment of women			
Maternity “high-care” ward, postnatal ward and gynecology ward visits	• Identified eligible women • Allowed a personal face-to-face approach	• Good for identification of potential future participants but not good for (immediate) recruitment as most postnatal women were already using long-acting contraception and were, therefore, temporarily ineligible • Potential participants were often unwell or traumatized, therefore, were not ready to be engaged with information on future research and pregnancy • There were also ethical considerations, as women were vulnerable, some after having had a near-death experience or having lost their baby • Recruiters needed permission to enter wards and sometimes felt like they were being a nuisance • The process was time-consuming: ward registers were often incomplete so it was necessary to “trawl through the entire ward looking at each bed-letter” • Eligible women delivering during the weekend could be missed • Recruiters found it difficult to interest ward staff, including doctors, in identifying potential participants (few women were recruited by such referrals)	• Establish a good relationship with ward staff to facilitate access to current ward registers and bed-letters and keep staff updated on the trial progress, e.g., by arranging meetings with them • Identify key staff members who will notify recruiters about potential participants, ideally on a daily basis, and especially about those women who deliver and are discharged during the weekend. Shared online spreadsheets can be useful for this purpose • Subsequent recruitment is most likely to be successful if the potential participant has met the recruiter previously face-to-face • Establish a support network for women with newborn loss to help maintain contact with potentially eligible postnatal women, and to facilitate discussion about the trial at a later date • Have a system in place to sort temporarily ineligible women according to methods of contraception (with date of next injection, if applicable) so that the timing of subsequent attempt/s to recruit are appropriate • During subsequent calls to elicit participants, recruiters should be aware of the outcome of the recent pregnancy, particularly in the event of stillbirth, and should be mindful of the woman’s emotional needs
Antenatal ward visits	• Identified future eligible women • Allowed a personal face-to-face approach	• Women in the antenatal wards were temporarily ineligible for a potentially long period before being eligible • It was easy to duplicate entries of potential participants	• Have a system in place to sort temporarily ineligible women according to gestational age at initial contact so that the timing of subsequent attempt/s to recruit are appropriate (e.g., 3-monthly intervals) • Marking patient folders (e.g., with a highlighter or sticker) once they have been identified reduces recruiter effort and the risk of duplicate entries
Postnatal clinic and gynecology outpatient clinic visits	• Identified women keen to engage with the health system regarding future pregnancy and usually willing to participate • Allowed a personal face-to-face approach • Good recruitment source	• Minimal difficulties were noted with this approach, which facilitates immediate recruitment	• Establish a good relationship with clinic staff to facilitate notification about potentially eligible women, ideally on a daily basis • Recruiter “business” cards and flyers for women to take home were useful with this approach • Consider ways to incentivize clinic staff and keep them updated about trial progress
Baby clinic visits	• Identified eligible women • Personal face-to-face approach	• Poor response/recruitment • Many women attending “high-care” baby clinics said they did not want another baby	• Probably not a good use of resources
Other outpatient departments and pharmacy waiting-rooms	• Identified some eligible women • Waiting-rooms have a “captive audience”	• People in these settings (particularly where there were long queues) often appeared anxious and impatient to have their needs attended to, so “were not interested in listening” to research staff. One recruiter stated that “they were very bored and noisy” • Yields with this method were low	• This approach might work best in settings with a dedicated pharmacy waiting-room or queue for women

Abbreviations: *LHW* lay health worker, *LMICs* low- and middle-income countries, *PE/E* pre-eclampsia/eclampsia

Prospective visits to wards and clinics were also very time-consuming. Eligible recruits were fewer than with the retrospective method, with many women being temporarily ineligible. However, yields were higher, probably due to the personal, face-to-face approach, which facilitated better communication and time for discussion with those women who were interested. Women recruited with this method represented potential “future” rather than immediate recruits, and time needed to be invested with regular follow-up telephone calls during their “ineligible” phase. This probably contributed to the longer-than-expected trial duration.

In total, 2563 women were screened and 1354 eligible non-pregnant women were recruited and randomized. Participant characteristics stratified by country can be found in Table 2. The median time since participants’ last pre-eclampsia-complicated pregnancy was 10.5 months (interquartile range (IQR) 29.4). Among participants recruited from African sites, poor previous pregnancy outcomes were common with more than half of African participants having experienced a fetal death, and more than a quarter having experienced eclampsia and/or HELLP (hemolysis, elevated liver enzymes and low platelets) syndrome, in their last pregnancy.

**Table 2:** Baseline characteristics and previous pregnancy outcomes of randomized non-pregnant women (*n* = 1354)

Country	Baseline characteristics							Previous pregnancy outcomes							
	Age		Parity		Time since last complicated birth (months)			Baby born alive		Gestational age at delivery		Eclampsia and/or HELLP syndrome		Onset of pre-eclampsia (weeks)	
	*n*	Mean [SD]	*n*	Mean [SD]	*n*	Mean [SD]	Median (IQR)	*n*	%	*N*	Mean [SD]	*n*	%	*n*	Mean [SD]
Argentina	117	29.4 [7.3]	117	1.6 [1.0]	117	15.7 [17.8]	8.9 (15.0)	111	94.9	116	35.2 [4.0]	21	18.1	111	32.6 [5.3]
South Africa	955	30.2 [5.7]	955	1.9 [1.0]	909	25.6 [37.2]	10.4 (33.5)	467	48.9	765	30.5 [6.1]	246	29.9	655	28.0 [6.5]
Zimbabwe	282	30.7 [5.6]	282	2.3 [1.3]	266	20.7 [24.8]	11.5 (18.7)	107	37.9	277	30.9 [5.5]	66	24.4	267	27.9 [5.7]
Total	1354	30.3 [5.9]	1354	2.0 [1.1]	1292	23.7 [33.7]	10.5 (29.4)	685	50.6	1158	31.1 [6.0]	333	27.5	1033	28.4 [6.3]

Abbreviations: *IQR* interquartile range, *n* number of participants, *SD* standard deviation, *HELLP* hemolysis, elevated liver enzymes and low platelets

Excluding the short first and last recruitment years, the average annual recruitment over the 4-year period from 2012 to 2015 was 298 participants per year (range 218 to 369). Overall, approximately one out of every two women screened was recruited; however, the proportion of women recruited out of those screened increased over time (Table 3).

**Table 3:** Annual number of women screened and randomized per country^a^

	Argentina		South Africa		Zimbabwe		Total	
Year	S	R	S	R	S	R	S	R
2011	0	0	41	33 (80.5%)	0	0	41	33 (80.5%)
2012	0	0	347	143 (41.2%)	300	75 (25.0%)	647	218 (33.7%)
2013	92	50 (54.3%)	319	179 (56.1%)	161	77 (47.8%)	572	306 (53.5%)
2014	55	31 (56.4%)	489	280 (57.3%)	103	58 (56.3%)	647	369 (57.0%)
2015	43	36 (83.7%)	368	217 (59.0%)	74	45 (60.8%)	485	298 (61.4%)
2016	0	0	129	103 (79.8%)	42	27 (64.3%)	171	130 (76.0%)
Total (%)	190	117 (61.6%)	1693	955 (56.4%)	680	282 (41.5%)	2563	1354 (52.8%)

Abbreviations: *S* screened, *R* randomized (the numbers in brackets is the percentage recruited of those screened)

^a^Recruitment commenced in South Africa in July 2011, in Zimbabwe in January 2012, and in Argentina in February 2013

Approximately 78% (961/1231) of randomized participants attended their first pre-pregnancy visit (PPV), theoretically 12 weeks after randomization, and participant attendance was above 86% for the second PPV (709/821) and all subsequent PPVs. However, timely attendance (within 2 weeks of the scheduled date) was lower, ranging between 57% (698/1231 at PPV 1) and 83% (24/29 at PPV 14), with higher compliance observed over time.

By September 2016, 45.3% of participants (614/1354) had become pregnant with 33.5% (454/1354) of participants becoming pregnant within 1 year of randomization, representing 73.9% (454/614) of all pregnancies. The total number of woman-years of follow-up to achieve one pregnancy was 3.7 woman-years, and to achieve a valid outcome (defined as delivery of a live child or miscarriage or stillbirth with gestational age of 20 weeks or more) was 4.8 woman-years.

## Discussion

To our knowledge, this is the first study of recruitment and retention strategies for preconception trials in LMICs. A range of recruitment approaches was used in this trial that built upon our experiences and lessons learnt from previous RCTs conducted among pregnant women [21, 23]. The majority of participants were recruited following identification from medical records/registers or from ward/clinic-based strategies, which enabled the accrual of a target sample of highly eligible women. However, the slower-than-anticipated accrual and the difficulties experienced by recruiters highlights that many LMIC trial procedures can be improved upon, in particular, by the deployment of more trial staff dedicated to specific recruitment and retention activities and the use of modern technology to facilitate these activities.

A report of a United States trial on preconception low-dose aspirin that used provider/clinic and community-based outreach recruitment approaches concluded that the former was most successful and cost-effective strategy [24]. Unfortunately, in our trial the source of recruits was not prospectively recorded; therefore, it was not possible to correlate recruitment approach with effectiveness. However, it is evident from the qualitative data that certain tasks, particularly the process of identifying and contacting women through hospital records and databases, were very laborious. One of the challenges for recruiters was the high proportion of redundant contact details and inaccurate recorded diagnoses leading to the inability of recruiters to contact many potentially eligible participants and to their incorrect identification and eligibility status. Retrospectively searching records to 5 years was probably too far back to search in our settings and searching a shorter period might have yielded similar participant numbers with a reduction in time invested. Few participants were recruited by self-referral in response to advertising or community outreach strategies. Researchers at one South African site reported a good response following promotion of the trial on a radio talk-show, and this and/or radio advertising may be a worthwhile option to consider in future trials, particularly as radio is an enduring and ubiquitous media in developing countries [25]. Women who refer themselves might also be more motivated to comply with trial visits than those recruited by other methods.

As anticipated, attendance at PPVs was not ideal and recruiters struggled to keep track of telephone contacts and visit schedules for both potential and randomized participants. Such difficulties occurred partly as a result of over-enthusiastic recruitment initially, whereby some women who probably were not particularly interested in the trial were recruited. Over time, however, recruitment and retention improved as recruiters learnt to identify those women genuinely interested in participating, and to pace recruitment efforts against existing follow-up visits. This evolved approach probably accounts for the improved recruitment rate over time. Concerning retention, one South African site reported that mobile phone applications (e.g., Whatsapp) were valuable for following up participants who missed visits as, with such applications, it was possible to see whether a telephone number was current and if the participant had read the message; it was also less time-consuming than a telephone call. In future, to better facilitate recruitment and retention processes, customization of appropriate mobile phone and web-based electronic tools (calendars, diaries) for eligibility screening, follow-up of temporarily ineligible women, and for scheduling follow-up visits and telephone calls with study participants would be a worthwhile investment in LMIC settings, as well as pre-trial recruiter training.

The high proportion of participants recruited through maternity wards and clinics probably contributed to the prolonged accrual time. Most postnatal women at the African sites are offered long-acting hormonal contraception before discharge and such women would have been identified as temporarily ineligible and followed up at 3-monthly intervals thereafter. Routine pre-pregnancy counseling as practiced in certain countries, such as China, helps to facilitate participant recruitment in preconception trials [10], and recruitment would no doubt have been easier if the CAP trial sites offered routine pre-pregnancy counseling for women with previous pre-eclampsia. However, even with this strategy, a certain proportion of eligible women would be missed because so many pregnancies in our settings are unplanned.

True to our sample size calculation, about half of the recruited women fell pregnant. As most pregnancies (73.9%) occurred within 12 months of randomization, investigators may wish to consider the cost-effectiveness of following up non-pregnant women beyond this or another time-point. In addition, when the planned stopping date is in sight, e.g., 6 months away, it would be prudent to have a strategy in place to stop follow-up of non-pregnant women to avoid investing unnecessary effort.

## Conclusions

This sub-study highlights that the most important resources for effective recruitment and retention in preconception trials are motivated and trained human resources. Searching and screening eligible participants who are not yet identifiable by the occurrence of a pregnancy is a very laborious process. However, with a combination of retrospective and prospective approaches, it is possible to yield a sample of highly eligible non-pregnant women. Investigators and funders of future preconception trials in low-resource settings should budget for sufficient on-site researchers and pre-trial researcher training to optimize recruitment and retention. In addition, they should consider using mobile phone and web-based electronic tools. Such deployment should lead to greater recruitment and retention efficiency and shorter trial durations.

## Abbreviations

CAP: Calcium And Pre-eclampsia
HELLP: Hemolysis, elevated liver enzymes and low platelets
IQR: Interquartile range
LMIC: Low- and middle-income country
PPV: Pre-pregnancy visit
RCT: Randomized controlled trial
WHO: World Health Organization
